# Amphiphilic Covalent Organic Framework for Efficient DHT Adsorption and Androgenetic Alopecia Treatment via Spectral Technology

**DOI:** 10.3390/molecules31162936

**Published:** 2026-08-21

**Authors:** Jiahui Wei, Fanqiang Bu, Bing Zhao, Qi Liu, Jinqiang Wu

**Affiliations:** 1 Heilongjiang Provincial Key Laboratory of Surface Active Agent and Auxiliary, Chemistry and Chemical Engineering Institute, Qiqihar University, Qiqihar 161006, China; 2 School of Basic Medical Sciences, Changzhi Medical College, Changzhi 046000, China

**Keywords:** covalent organic frameworks, amphiphilicity, dihydrotestosterone, androgenetic alopecia

## Abstract

Androgenetic alopecia (AGA) is a prevalent clinical disorder, and the key pathogenic factor is dihydrotestosterone (DHT) present in the pilosebaceous unit. Current clinical therapeutic options are often associated with notable adverse effects, highlighting the urgent need for safer and more effective interventions. Herein, a novel amphiphilic covalent organic framework (amCOF) is designed and synthesized. By simultaneously incorporating hydrophilic functional groups and lipophilic alkyl chains into the covalent organic skeleton, the material exhibits excellent amphiphilicity, high specific surface area, and well-ordered porous structures. Owing to its amphiphilic nature, amCOF disperses uniformly in aqueous physiological media and adapts favorably to the lipophilic microenvironment within hair follicles. Furthermore, the ordered porous architecture endows amCOF with rapid DHT adsorption kinetics, high adsorption capacity, and high removal efficiency. Functional assays demonstrate that amCOF effectively reverses the inhibitory effects of DHT on the proliferation and migration of human dermal papilla cells. In animal models, topical application of amCOF significantly reduces local DHT concentrations, promotes hair regrowth, and shows favorable biosafety profiles. Collectively, this work provides a new strategy for treating androgenetic alopecia by scavenging pathogenic lipophilic molecules from sebum using amphiphilic porous materials and also establishes a solid foundation for expanding the biomedical applications of COFs.

## 1. Introduction

Androgenetic alopecia (AGA) is the most common type of alopecia in clinical practice [1,2], and its pathogenesis is closely associated with the conversion of testosterone to dihydrotestosterone (DHT) by 5*α*-reductase, which subsequently interferes with follicular metabolism [3,4]. Although drugs such as finasteride and minoxidil have been widely used in the clinical management of AGA, their therapeutic outcomes are often suboptimal, and relapse rates remain high after discontinuation [5,6]. Specifically, finasteride exerts its effects by inhibiting 5α-reductase activity but may cause sexual dysfunction and allergic reactions. Minoxidil promotes hair growth through local vasodilation, whereas propylene glycol, a constituent in its formulation, may induce local irritation and exert potential effects on blood pressure [7,8]. Notably, DHT tends to accumulate in the sebum secreted by the pilosebaceous glands, forming a biphasic “internal oil–external water” barrier that substantially impairs the elimination efficiency of conventional hydrophilic drugs against DHT [9,10]. Therefore, how to achieve efficient local clearance of DHT while circumventing the systemic side effects of existing drugs has become a central challenge in the development of AGA therapeutic strategies.

In recent years, various emerging delivery strategies have been explored to improve the therapeutic efficacy of AGA-related agents, including magnetic actuation, optical actuation, catalytic effects, microneedle delivery, enzymatic degradation, and physicochemical modulation of nanoparticle structures [10,11]. Although these strategies have made certain progress in enhancing drug bioavailability, their primary mode of action remains focused on facilitating the delivery and release of exogenous drugs rather than directly targeting the pathogenic molecule DHT itself [12,13]. By contrast, direct removal of overexpressed DHT from the follicular microenvironment holds the potential to intervene in the disease process at its origin and fundamentally circumvent drug-induced hormonal imbalance [14,15]. However, functional materials capable of achieving precise and efficient in situ clearance of DHT within hair follicles are still lacking. A critical prerequisite for realizing this goal is that the material must maintain satisfactory dispersibility and interfacial affinity in both aqueous and oil phases so as to effectively overcome the sebum barrier [16,17]. Moreover, given that DHT is also one of the hormones essential for normal physiological metabolism, another major challenge lies in achieving selective removal of locally excessive DHT within hair follicles, rather than systemic depletion.

Covalent organic frameworks (COFs) are a class of porous crystalline materials constructed from organic monomers via covalent bonds, and they possess unique advantages, including pre-designable structures, high specific surface areas, well-ordered pore channels, and ease of functional modification, thereby demonstrating broad application prospects in adsorption, separation, and drug delivery in recent years [18,19]. Owing to the high tunability of COF structures, several studies have successfully constructed amphiphilic COFs by simultaneously introducing hydrophilic and hydrophobic groups into the framework [20,21]. Such materials not only retain the high porosity and structural stability of COFs but also exhibit favorable dispersibility and interfacial affinity in both aqueous and oil phases, thus providing a feasible platform for capturing specific bioactive molecules in complex physiological environments [22,23]. Although amphiphilic COFs have gradually attracted attention in the fields of interface science and drug delivery, their biomedical applications, particularly in the targeted elimination of specific pathological molecules within the follicular sebum microenvironment, remain in their infancy [24,25]. To date, no study has reported the use of amphiphilic COFs for adsorbing DHT from sebum to treat AGA, and existing materials still face significant challenges in simultaneously achieving high adsorption capacity, excellent affinity, and satisfactory biocompatibility, necessitating rational material design and functional optimization tailored to the molecular structure of DHT and the characteristics of the sebum microenvironment.

Herein, a novel amphiphilic covalent organic framework (amCOF) was designed and synthesized, with the aim of utilizing its amphiphilic properties to achieve precise in situ adsorption of overexpressed DHT within hair follicles, thereby attaining therapeutic effects against AGA. The amCOF was endowed with excellent amphiphilicity by simultaneously incorporating hydrophilic functional groups and lipophilic alkyl chains into the covalent organic skeleton. Microscopic structural characterization confirmed the amphiphilic nature, structural stability, high specific surface area, and well-ordered pore architecture of amCOF. Adsorption kinetic and isotherm studies further demonstrated that amCOF possesses high efficiency in adsorbing and removing DHT. In simulated sebum, water interface environments, and cellular assays, amCOF effectively relieved the inhibitory effect of DHT on the migration of human dermal papilla cells following DHT clearance. In vivo animal experiments revealed that topical application of amCOF significantly reduced local DHT concentrations, promoted hair regrowth, and exhibited favorable biosafety profiles. Collectively, this study provides a new strategy for the targeted elimination of pathogenic molecules from sebum using amphiphilic porous materials in the treatment of AGA and also lays an experimental and theoretical foundation for expanding the biomedical applications of covalent organic frameworks.

## 2. Results and Discussions

### 2.1. Synthesis and Characterization

**Figure 1:**
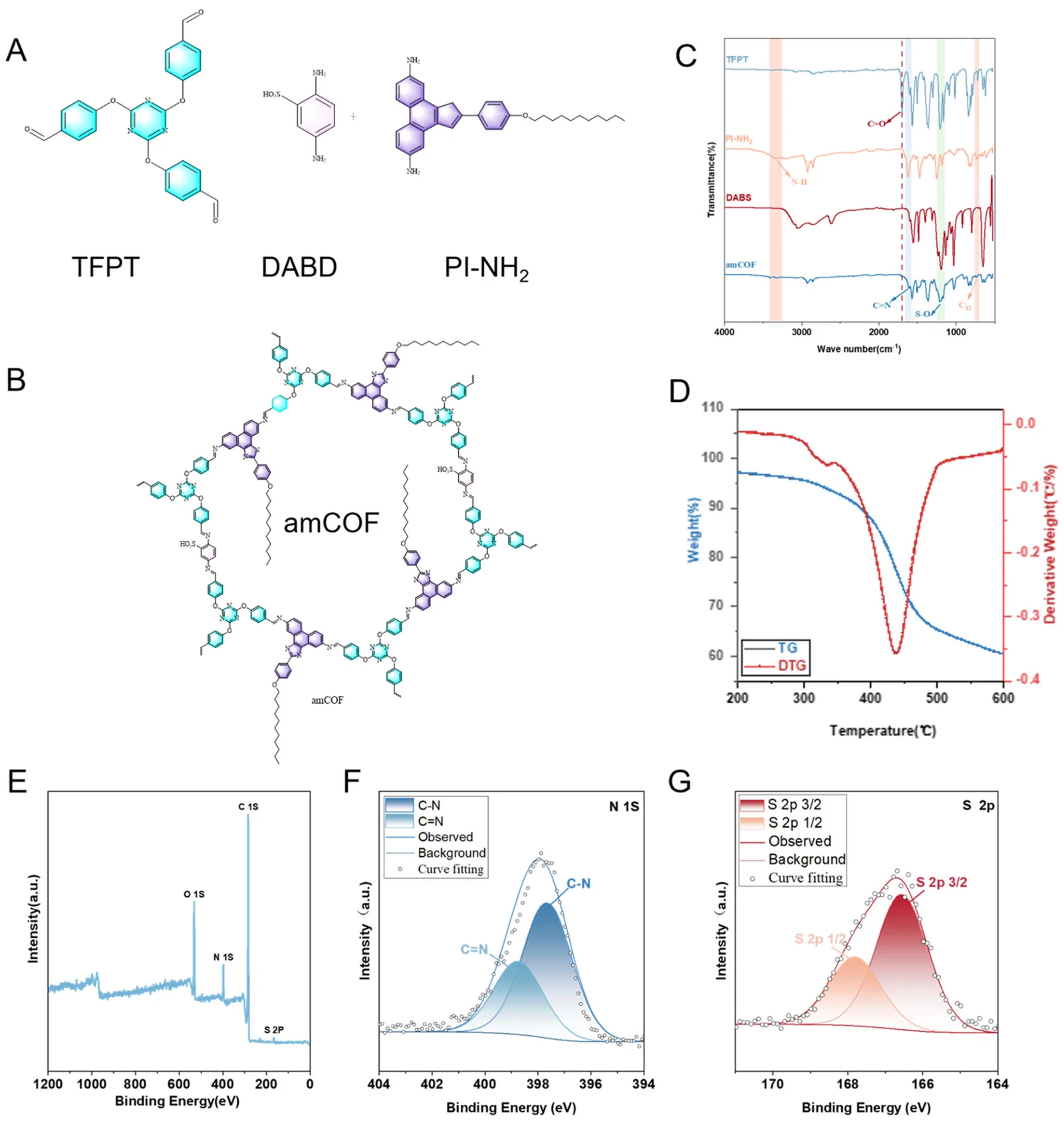
Structure and characterization of COF: (**A**) Chemical structures of the monomers. (**B**) Schematic illustration of the amCOF structure. (**C**) FTIR spectra of the monomers and amCOF. (**D**) TGA and DTG curves of amCOF. (**E**) Survey XPS spectrum of amCOF. (**F**) High-resolution N 1s XPS spectrum of amCOF. (**G**) High-resolution S 2p XPS spectrum of amCOF.

The chemical structures of the three monomer derivatives used for the construction of the amCOF were confirmed by ^1^H NMR and ESI-MS (Figure 1A and Figures S1–S4). The chemical structure of amCOF is illustrated in Figure 1B. In the Fourier-transform infrared (FT-IR) spectra (Figure 1C), the characteristic absorption band of the C=O bond in the TFPB monomer at 1701 cm^−1^, together with the broad band of the N–H bond in the PI-NH_2_ monomer in the range of 3200–3400 cm^−1^, weakened significantly. Concurrently, a new characteristic absorption band attributed to the imine bond (C=N) appeared at 1611 cm^−1^. This result indicated that the Schiff base condensation reaction between the monomers had proceeded successfully, thus directly confirming the successful synthesis of amCOF. In addition, the absorption band observed at approximately 730 cm^−1^ was assigned to the rocking vibration of the –CH_2_ groups in the long alkyl chains, suggesting that the lipophilic alkyl chains had been successfully introduced into the COF skeleton, which further corroborated the successful construction of amCOF. The thermal stability of amCOF was evaluated by thermogravimetric analysis (TGA) and derivative thermogravimetric analysis (DTG). Only a slight weight loss was observed in the temperature range from room temperature to 400 °C, which was attributable to the volatilization of solvent molecules adsorbed in the pores and the decomposition of a small amount of unreacted low-molecular-weight compounds (Figure 1D). When the temperature exceeded 400 °C, a significant mass loss occurred, indicating the collapse and thermal decomposition of the framework. The decomposition temperature of the framework was thereby determined to be approximately 438 °C. Upon heating to 1000 °C, the total weight loss of the material stabilized at approximately 42%, revealing a relatively high carbon residue rate. Collectively, these results demonstrated that amCOF possessed good thermal stability, which laid a foundation for its subsequent application in complex physiological environments.

To further confirm the chemical composition and bonding structure of amCOF, X-ray photoelectron spectroscopy (XPS) was employed (Figure 1E). The survey XPS spectrum revealed four characteristic peak series with binding energies located at 284.08 eV (C 1s), 398.08 eV (N 1s), 532.08 eV (O 1s), and 166.08 eV (S 2p), indicating that the material was composed of carbon, nitrogen, oxygen, and sulfur, which were fully consistent with the theoretical elemental composition of its topological structure. In the high-resolution N 1s XPS spectrum (Figure 1F), two distinguishable characteristic peaks were observed at 397.68 eV and 398.78 eV, which were assigned to C–N and C=N bonds, respectively. The appearance of the C=N peak provided direct evidence for the formation of imine bonds via the Schiff base condensation reaction, confirming the successful construction of the imine-linked network in amCOF. Meanwhile, the C–N peak was attributed to the nitrogen atoms within the phenanthroimidazole structural units, which was in good agreement with the expected structure. In the high-resolution S 2p XPS spectrum (Figure 1G), a characteristic doublet arising from spin–orbit splitting was observed at 167.78 eV and 166.58 eV, which were assigned to S 2p_1/2_ and S 2p_3/2_, respectively. The clear emergence of the sulfur-related peaks confirmed that the sulfonic acid groups had been successfully introduced into the amCOF framework. Collectively, the XPS results demonstrated that the target material amCOF had been successfully constructed via the Schiff base condensation reaction among the long-chain alkyl-containing monomer, TFPB, and the sulfonic acid-containing monomer, with its chemical composition being fully consistent with the expected structure.

### 2.2. Crystal and Morphology

**Figure 2:**
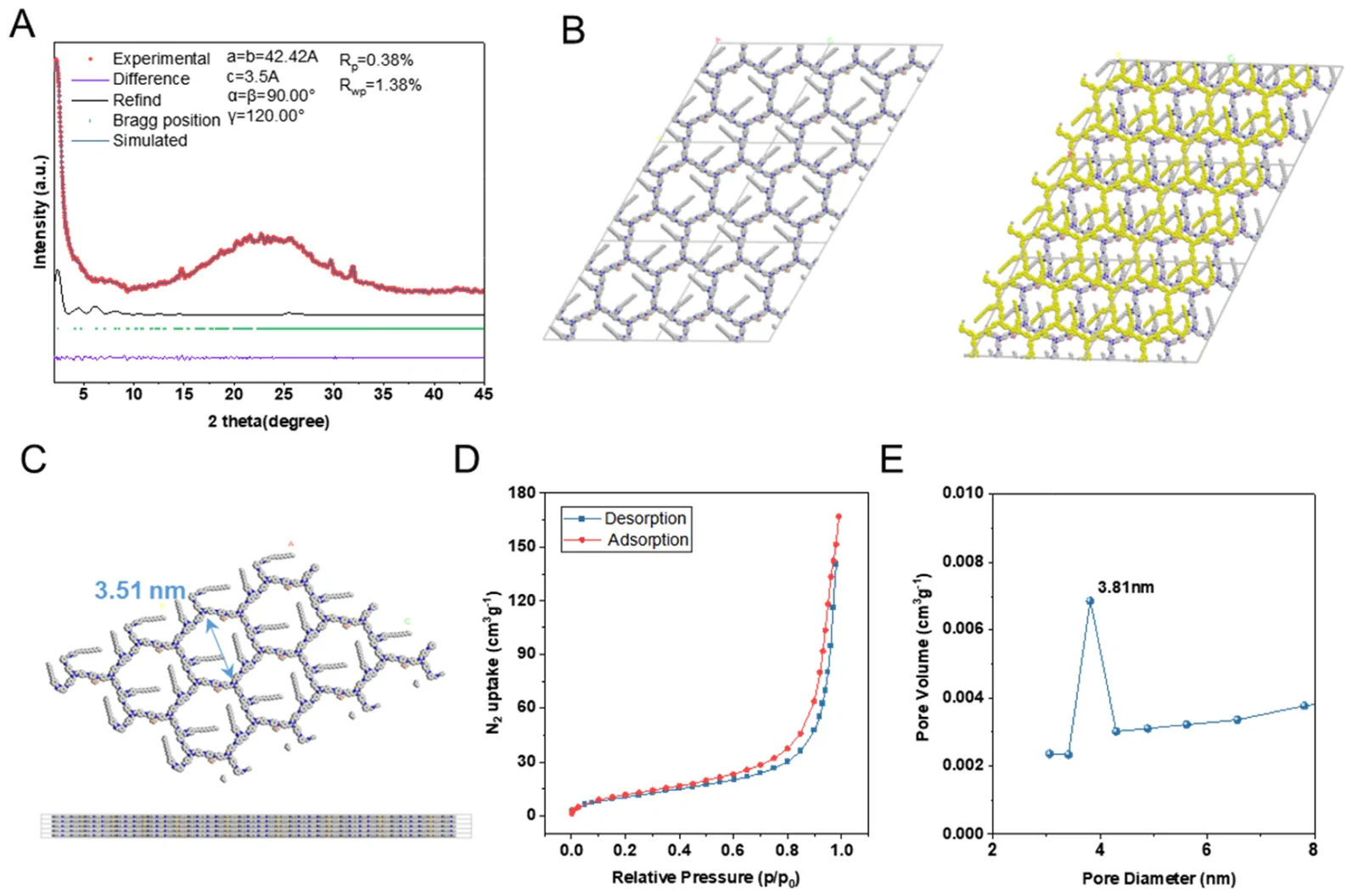
Crystalline and packing characterization of amCOF: (**A**) PXRD pattern of amCOF. (**B**) Simulated AA-stacked and AB-stacked modes of amCOF. (**C**) Pore size and interlayer spacing of amCOF in the AA-stacked model. (**D**) N_2_ adsorption–desorption isotherm of amCOF. (**E**) Pore size distribution of amCOF.

Powder X-ray diffraction (PXRD) patterns revealed three characteristic diffraction peaks for amCOF at approximately 2.4°, 4.1°, and 23.0° (Figure 2A). The strong diffraction peak at 2.4° was assigned to the (100) crystal plane, while the weak peak at 4.1° corresponded to the (110) crystal plane, indicating that the material possessed a long-range ordered crystalline structure. To further elucidate the interlayer stacking mode, structural simulations were performed using Material Studio software (2024) for both AA-stacked and AB-stacked configurations (Figure 2B). The experimentally obtained PXRD pattern was compared with the two simulated stacking modes. The results demonstrated that the experimental pattern was in excellent agreement with the AA-stacked simulation, whereas a notable deviation was observed with the AB-stacked mode, thereby confirming that amCOF adopted an AA stacking arrangement. The consistency between the experimental and simulation results suggested that this stacking mode was likely driven by the rigid, planar nature of the phenanthro[9,10-d]imidazole structural units in the PI-NH_2_ monomer, which facilitated ordered interlayer π–π stacking interactions in the AA-stacked configuration. Molecular simulations further revealed that the TFPB and PI-NH_2_ units in amCOF were connected via covalent bonds to form closed regular hexagonal rings, with a theoretical pore size of approximately 35.1 Å (Figure 2C). In the AA-stacked mode, the interlayer distance between adjacent monolayers was determined to be approximately 3.5 Å, which was consistent with typical π–π stacking distances, further supporting the tightly packed structural model. The above structural analyses indicated that the regularly aligned hexagonal units constituted the smallest repeating motif of amCOF, which extended to form the overall topological network, and the final structure of amCOF was found to be in excellent agreement with the initial design expectations. The porous characteristics of amCOF were evaluated by nitrogen adsorption–desorption isotherms (Figure 2D). The isotherm exhibited a type IV curve with an H3-type hysteresis loop, indicating that the material possessed a typical mesoporous structure with relatively ordered pore channels. The specific surface area of amCOF was calculated to be 167.3 m^2^·g^−1^ using the BET model. Notably, this relatively low specific surface area was possibly attributed to the occupation of pore spaces by the long alkyl chains introduced into the framework, which reduced the accessible surface area for nitrogen molecules. The average pore diameter was estimated to be approximately 3.81 nm by the BJH method (Figure 2E), which was close to the theoretical pore size, further validating the rationality of the structural model. Collectively, the combined results of crystal structure analysis and porous characterization confirmed the successful synthesis of the target material amCOF, which exhibited high crystallinity, well-ordered mesoporous channels, and good thermal stability, thereby establishing a solid material foundation for subsequent adsorption applications.

**Figure 3:**
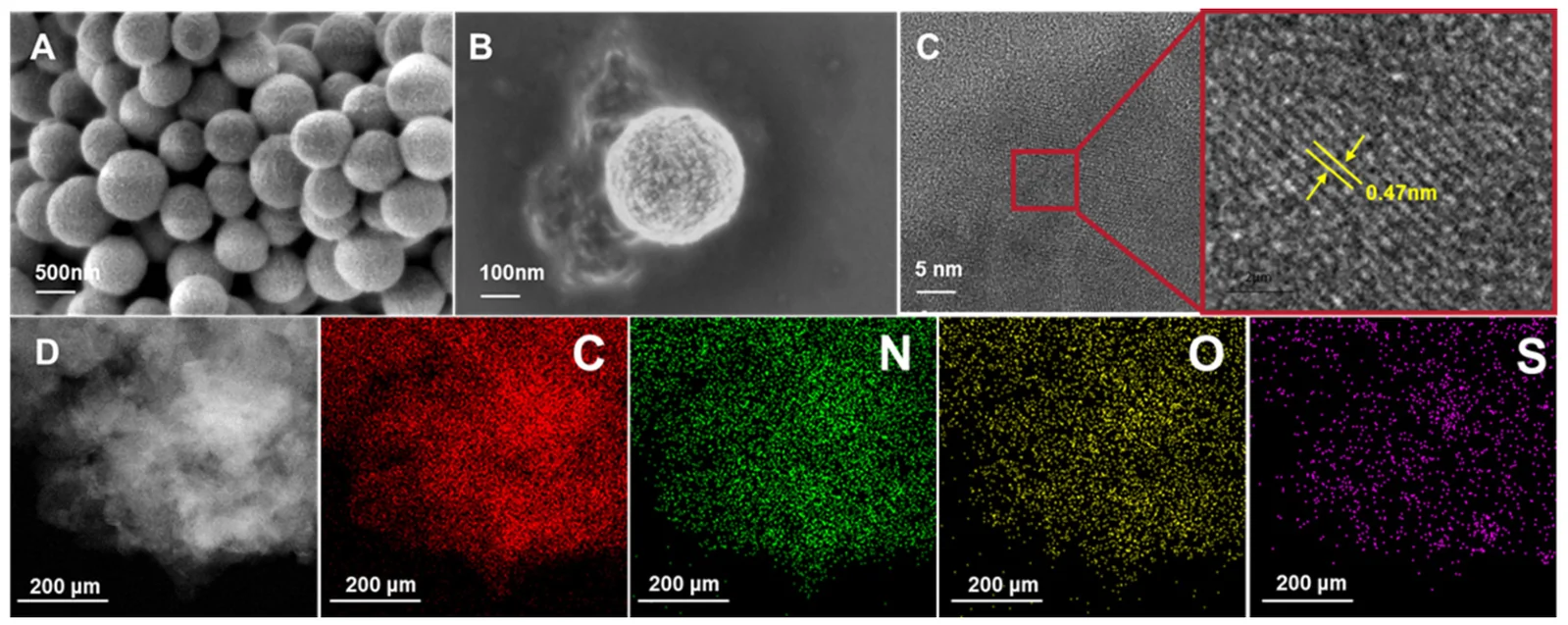
Morphological characterization of amCOF: (**A**) SEM image of amCOF, scale bar = 500 nm. (**B**) SEM image of amCOF, scale bar = 100 nm. (**C**) HR-TEM image of amCOF, scale bar = 5 nm. (**D**) EDS elemental mapping of amCOF, scale bar = 200 nm.

The microstructure and crystalline features of amCOF were systematically characterized by scanning electron microscopy (SEM), transmission electron microscopy (TEM), and high-resolution transmission electron microscopy (HR-TEM). SEM images revealed that amCOF exhibited regular spherical particles with an average diameter of approximately 230 nm (Figure 3A,B). HR-TEM imaging clearly resolved the lattice fringes of amCOF (Figure 3C), and the measured interplanar spacing corresponding to the (100) crystal plane was approximately 0.47 nm, further corroborating the highly crystalline nature of the material. A minor discrepancy was observed between the experimentally determined lattice spacing and the values predicted by molecular simulation, which was likely attributable to the differences between the idealized models used in the simulations and the actual synthesized samples, where local defects, surface effects, or residual stresses may inevitably exist. Energy-dispersive X-ray spectroscopy (EDS) elemental mapping revealed that C, N, O, and S were uniformly distributed throughout the amCOF framework (Figure 3D), further confirming the chemical homogeneity and structural integrity of the material. Collectively, these physicochemical characterization results demonstrated that amCOF possessed well-ordered arrangements and excellent crystallinity. Moreover, the mutual consistency between the experimental data and theoretical simulations validated the rationality of the initial structural design, thereby establishing a solid material foundation for subsequent application studies.

### 2.3. Adsorption Performance of amCOF Toward DHT

**Figure 4:**
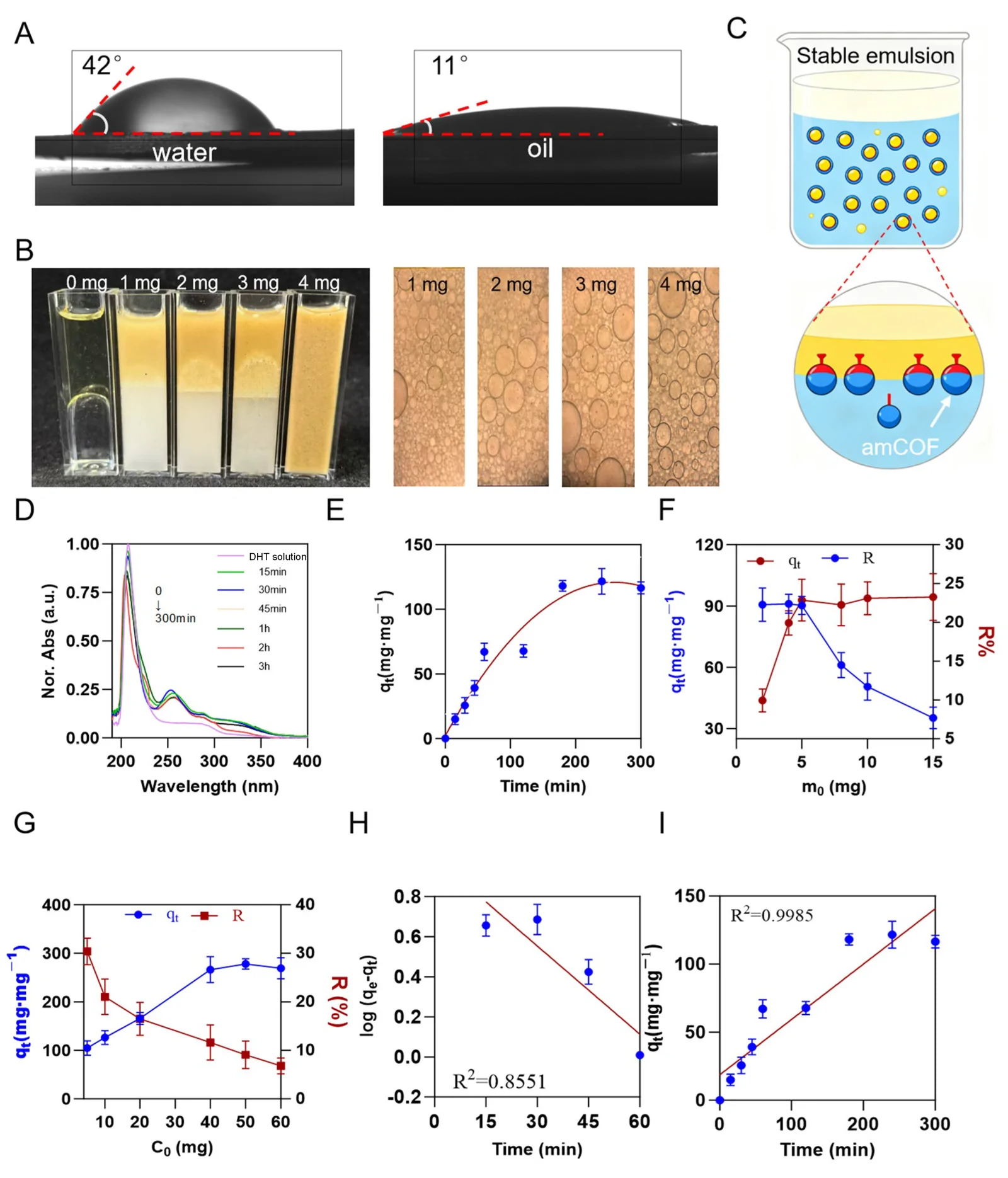
Amphiphilic properties and DHT adsorption performance of amCOF: (**A**) Water and oil contact angles of amCOF. (**B**) Emulsification performance of amCOF toward a typical oil–water system. (**C**) Schematic illustration of the amphiphilic nature of amCOF. (**D**) UV–vis absorption spectra of DHT before and after adsorption by amCOF. (**E**) Adsorption capacity of amCOF toward DHT as a function of contact time. (**F**) Effect of amCOF dosage on the adsorption capacity and removal efficiency toward DHT. (**G**) Effect of initial DHT concentration on the adsorption capacity and removal efficiency of amCOF. (**H**) Pseudo-first-order kinetic fitting for DHT adsorption onto amCOF. (**I**) Pseudo-second-order kinetic fitting for DHT adsorption onto amCOF.

Subsequently, the surface wettability of the amCOF sample was evaluated by water contact angle measurements (Figure 4A). The water contact angle and oil contact angle of amCOF were determined to be 42° and 11°, respectively, indicating that the material possessed both hydrophilic and lipophilic properties, thereby exhibiting typical amphiphilic characteristics. The hydrophilic nature was primarily attributed to the introduction of sulfonic acid groups, whereas the lipophilic nature originated from the inherent hydrophobicity of the COF skeleton and the modification with long-chain alkyl functional groups. These results demonstrated that the surface wettability of COF materials could be precisely tailored through rational design and modulation of the functional groups within the framework. Then, the amphiphilic interfacial activity of amCOF was further evaluated by testing its emulsification capability toward a typical oil–water two-phase system. Castor oil was selected as the oil phase, and deionized water was used as the aqueous phase. In the absence of amCOF, a distinct oil–water interface was observed (Figure 4B), indicating that the two phases were immiscible. With stepwise increases in the amount of amCOF added (1, 2, 3, and 4 mg, respectively), the emulsification phenomenon was progressively enhanced. Upon addition of 1 mg of amCOF, slight emulsification was observed in both the aqueous and oil phases, suggesting that amCOF effectively reduced the interfacial tension between the two phases. When the amCOF dosage reached 4 mg, the oil–water interface essentially disappeared, and a stable emulsion system was formed. Optical microscopy observations revealed that the emulsion droplets stabilized by amCOF exhibited a relatively broad size distribution, ranging from approximately 180 to 1000 μm. With increasing amCOF dosage, the number of oil-in-water emulsion droplets increased markedly, indicating that amCOF could serve as a solid emulsifier. The underlying emulsification mechanism could be attributed to the cooperative effects of the long-chain alkyl groups within the amCOF framework, which tended to anchor to the oil phase, and the sulfonic acid groups, which preferentially interacted with the aqueous phase. This synergistic action positioned amCOF at the oil–water interface, effectively reducing interfacial tension and forming stable emulsions (Figure 4C). Such amphiphilic properties also provided a robust theoretical basis for the uniform dispersion of amCOF within the dual environments of follicular sebum and physiological media.

One of the core challenges in the treatment of AGA lies in the effective elimination of DHT sequestered within follicular sebum; therefore, the adsorption performance of amCOF toward DHT was undoubtedly a central focus of this study. First, the absorbance of a series of DHT standard solutions with gradient concentrations was measured by UV-vis spectrophotometry, and the standard curve was constructed accordingly (Figure S5). Using 5.0 mg of amCOF as the adsorbent, the effect of adsorption time on its adsorption performance was investigated in the 100 mL DHT solution with an initial concentration of 20 mg·L^−1^. During the initial adsorption phase (0–60 min), the adsorption capacity exhibited a rapid increasing trend with time (Figure 4D). At 60 min, the adsorption capacity of amCOF toward DHT reached approximately 119.2 mg·g^−1^, approaching the equilibrium adsorption capacity. This high adsorption rate at the initial stage was attributable to the abundant unoccupied active sites on the amCOF surface and the relatively high DHT concentration in the solution, which together provided a sufficient mass transfer driving force, facilitating the rapid diffusion of DHT molecules to the material surface and subsequent adsorption. As the adsorption time was further extended to 60–180 min, the increase in adsorption capacity gradually slowed, entering a stage of slower growth (Figure 4E). When the adsorption time reached 180 min, the adsorption capacity approached equilibrium; further prolonging the adsorption time to 240 min did not result in any significant increase, indicating that the adsorption process had reached a dynamic equilibrium. Ultimately, after calculation, the equilibrium adsorption capacity of amCOF for DHT was approximately 118.45 mg·g^−1^. Subsequently, the effect of adsorbent dosage on DHT adsorption performance was investigated (Figure 4F). With increasing amCOF dosage, the equilibrium adsorption capacity (*q_e_*) exhibited a trend of rapid initial increase followed by leveling off, reaching a maximum of 117 mg·g^−1^ at a dosage of 5.0 mg, beyond which no significant change in *q_e_* was observed. The removal efficiency (*R*) showed an opposite trend: as the adsorbent dosage increased, *R* first increased and then decreased, with a maximum value of 24.71% also obtained at 5.0 mg. When the dosage exceeded 5.0 mg, the residual DHT concentration in solution became extremely low, resulting in a weakened mass transfer driving force. Meanwhile, the excess adsorbent particles may have undergone aggregation, which partially shielded the active sites, preventing the effective adsorption area from increasing proportionally with dosage and consequently leading to a decline in removal efficiency. As the initial DHT concentration increased from 5 to 60 mg·L^−1^, the equilibrium adsorption capacity *q_e_* first increased and then plateaued, reaching a maximum of 298 mg·g^−1^ at 40 mg·L^−1^ (Figure 4G). In contrast, the removal efficiency R exhibited a continuous decrease, with the highest value of 30.32% observed at 5 mg·L^−1^. These results indicated that the abundant adsorption sites at low DHT concentrations enabled a high removal efficiency, while the progressive saturation of these sites at higher concentrations led to an upper limit of adsorption capacity and a consequent reduction in removal efficiency. To elucidate the rate-controlling mechanism of DHT adsorption onto amCOF, the experimental data were fitted using the pseudo-first-order and pseudo-second-order kinetic models, respectively. The pseudo-first-order kinetic model yielded a correlation coefficient R^2^ of 0.8551 (Figure 4H), whereas the pseudo-second-order kinetic model gave a significantly higher R^2^ value of 0.9985 (Figure 4I).

To further elucidate the adsorption behavior and the nature of the adsorbent–adsorbate interaction, the equilibrium adsorption data were fitted to the Langmuir and Freundlich isotherm models. The Langmuir model assumes monolayer adsorption on a homogeneous surface with equivalent and independent binding sites, and it yielded a correlation coefficient of 0.99425 (Figure S6). By contrast, the Freundlich model, which describes multilayer adsorption on heterogeneous surfaces, gave a significantly lower R^2^ value of 0.97248 (Figure S7). This quantitative comparison clearly indicates that the Freundlich model fails to adequately describe the adsorption of DHT onto amCOF. The excellent fit to the Langmuir model strongly suggests that DHT molecules interact with amCOF at specific and energetically uniform binding sites, forming a monolayer. This finding provides critical thermodynamic evidence that complements the kinetic analysis. The combined results, namely the excellent fit to the pseudo-second-order kinetic model and the superior correlation with the Langmuir isotherm model, collectively point towards a chemisorption-dominated mechanism. The adsorption is therefore attributed to a synergistic effect of multiple specific interactions, including the hydrophobic effect driven by the lipophilic chains, strong π–π stacking between the electron-rich COF skeleton and the DHT steroidal core, and hydrogen bonding or electrostatic interactions from the sulfonic acid groups, all occurring on the well-ordered and uniform pore surfaces of amCOF.

### 2.4. AmCOF Reverses DHT-Induced Inhibition of hDPC Proliferation and Migration

**Figure 5:**
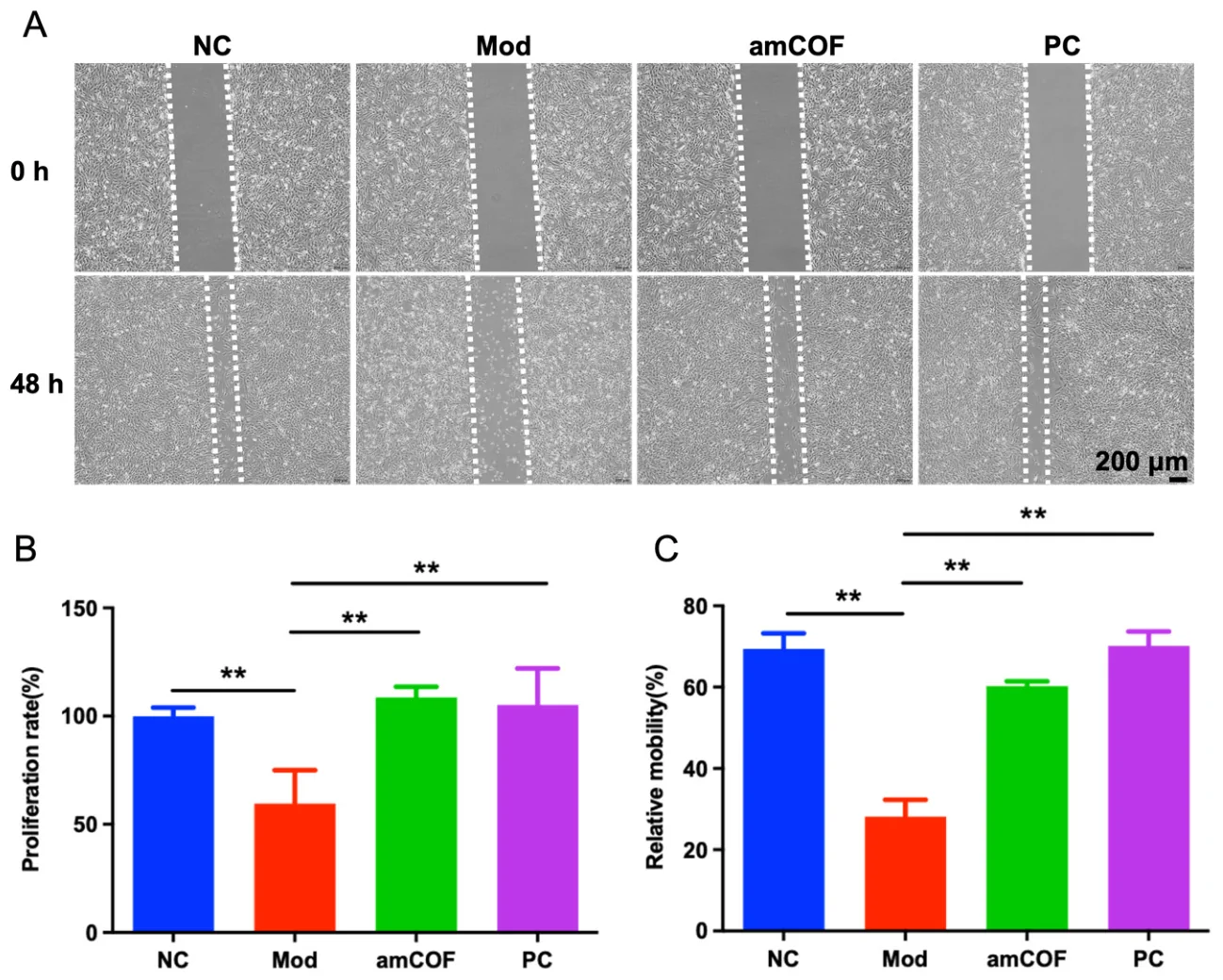
AmCOF reverses the inhibitory effects of DHT on the proliferation and migration of hDPCs: (**A**) Migration results of hDPCs in each group. Scale bar = 200 μm. (**B**) Proliferation rates of hDPCs in each group. (**C**) Relative migration efficiency of hDPCs in each group. *n* = 3; ** *p* < 0.01.

The proliferation and migration of human dermal papilla cells (hDPCs) are critical for maintaining the normal hair follicle growth cycle and regenerative capacity [26]. In this study, the untreated group was designated as the NC, and the minoxidil-treated group served as the PC. Compared with the NC group, DHT treatment significantly inhibited the proliferation and migration of hDPCs, suggesting that elevated DHT levels directly impaired the normal biological functions of dermal papilla cells, which was highly consistent with the core pathological mechanism of AGA (Figure 5A). DHT acted on dermal papilla cells by suppressing their proliferation and migration, thereby shortening the anagen phase of hair follicles and accelerating the miniaturization process, ultimately leading to hair loss. Upon amCOF intervention, the DHT-induced inhibition of cell proliferation and migration was significantly reversed, and the cellular biological activities were remarkably restored, with the recovery being comparable to that of the minoxidil-treated group. Quantitative analysis revealed that compared with the NC group, DHT treatment reduced the cell proliferation rate to 59%. After amCOF treatment, the cell proliferation rate increased to 108%, which was essentially consistent with that of the PC group (105%) (Figure 5B). Regarding cell migration, the relative migration efficiency in the NC group was 69%, while that in the DHT treatment group decreased to 28%. Upon amCOF treatment, the migration efficiency was restored to 60%, compared with 70% in the PC group; although slightly lower than that of the PC group, it was still significantly higher than that of the DHT treatment group (Figure 5C). These results demonstrated that amCOF effectively reversed the detrimental effects of DHT on hDPCs, restored normal cell proliferation and migration capacities, and thus exhibited promising therapeutic potential against androgenetic alopecia at the cellular level.

### 2.5. Therapeutic Evaluation and Histopathological Analysis of AGA

**Figure 6:**
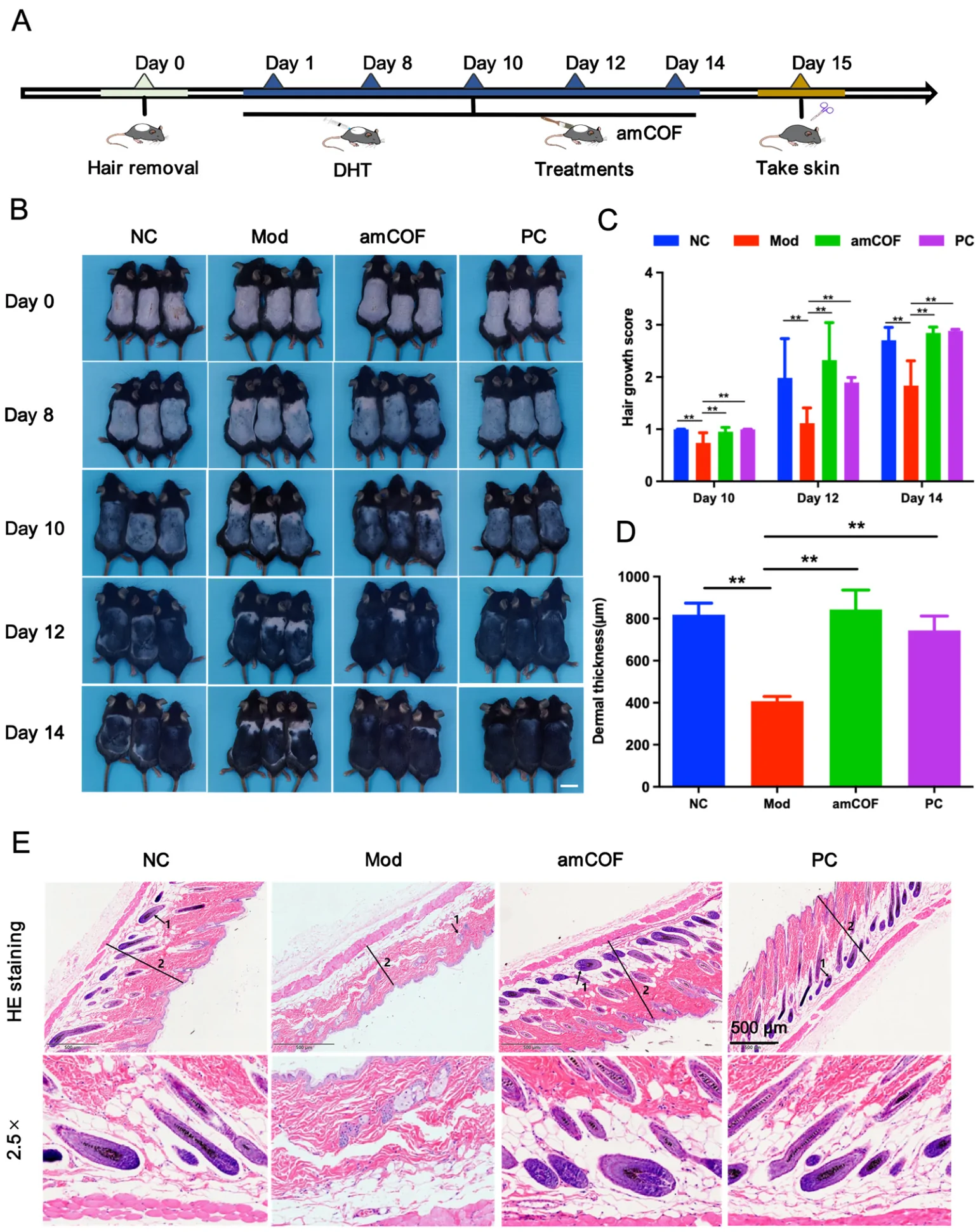
In vivo evaluation of AGA treatment by DHT adsorption onto amCOF: (**A**) Schematic illustration of the AGA model construction and treatment procedure. (**B**) Photographs of dorsal skin from mice in different treatment groups. Scale bar = 1 cm. (**C**) Relative hair regrowth area in different treatment groups. (**D**) Statistical quantification of dermal thickness in different treatment groups. (**E**) H&E-stained sections of mouse skin tissues from different treatment groups; 1: hair follicle; 2: dermal thickness. Scale bar = 500 μm. *n* = 8, ** *p* < 0.01.

To evaluate the in vivo DHT adsorption efficacy of amCOF, a *C57BL/6* mouse model of AGA was established [27]. Dorsal skin images of mice in different treatment groups were recorded on days 0, 8, 10, 12, and 14 after depilation to assess hair regrowth (Figure 6A). On day 0 post-modeling, the dorsal skin of all mice appeared uniformly pink without obvious pigmentation, indicating complete depilation and the synchronized telogen phase of hair follicles. From day 8 onward, pigmentation and hair shaft emergence were progressively observed (Figure 6B). On days 10, 12, and 14, hair regrowth in the DHT-induced group was markedly delayed, whereas amCOF treatment significantly ameliorated the inhibition of hair regeneration, with its hair-promoting effect being comparable to that of the positive control group (Figure 6C). These results indicated that amCOF effectively promoted hair regrowth in AGA model mice by adsorbing local DHT and optimizing the follicular microenvironment. Histological evaluation of mouse skin was further performed by H&E staining, assessing three parameters, including dermal thickness, hair follicle number, and hair follicle size. Compared with the NC group, DHT treatment significantly reduced dermal thickness, follicle number, and follicle volume, which decreased to 0.50-, 0.59-, and 0.41-fold of the NC group, respectively (Figure 6D). Notably, amCOF treatment significantly reversed these histological alterations, restoring dermal thickness, follicle number, and follicle size to levels comparable to those of the NC group (Figure 6E). Increased dermal thickness, elevated follicle number, and enlarged follicle size are important histological indicators of the transition of hair follicles from telogen to anagen phase (Figure S8). Histological analysis revealed that hair follicles in the DHT group remained predominantly in the telogen phase, whereas those in the NC, amCOF, and PC groups had entered the early-to-middle anagen phase (Figure S9). These findings suggested that DHT could maintain hair follicles in a prolonged telogen state, while amCOF effectively counteracted this inhibitory effect, driving the hair follicle cycle from telogen to anagen and consequently promoting hair regeneration.

**Figure 7:**
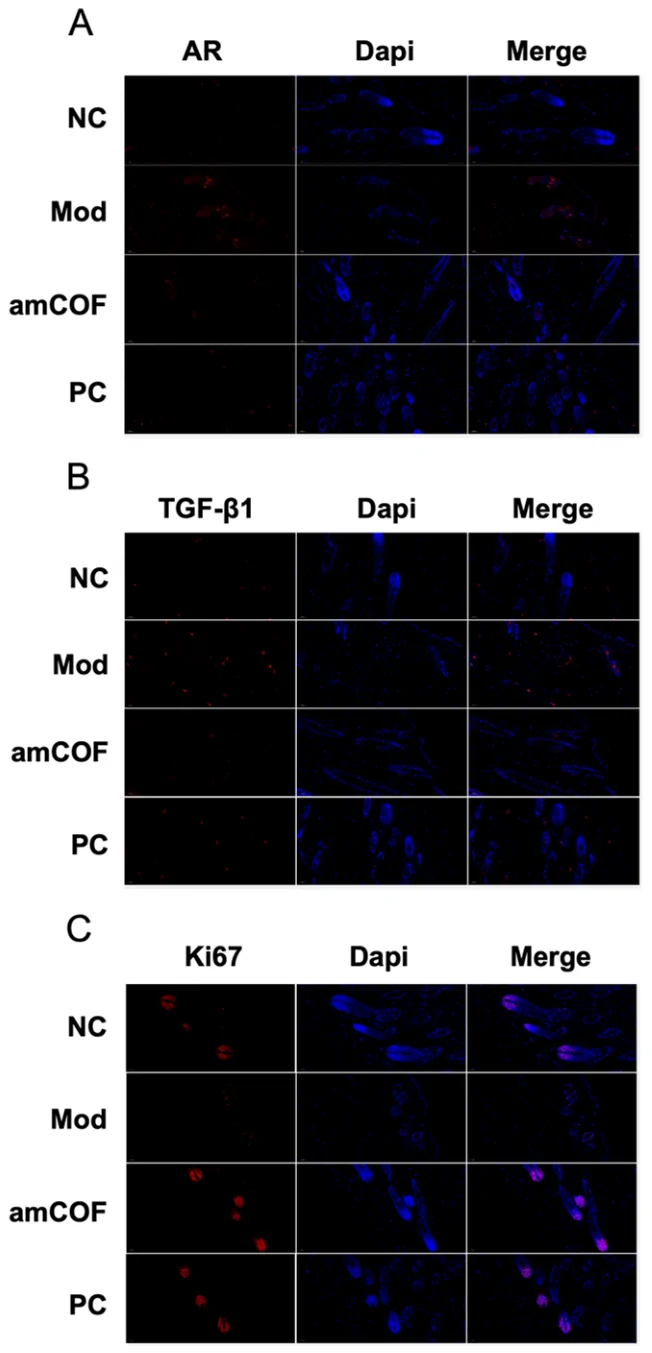
Immunofluorescence staining analysis of mouse skin sections from different treatment groups: (**A**) Immunofluorescence staining of AR (androgen receptor). (**B**) Immunofluorescence staining of TGF-β1. (**C**) Immunofluorescence staining of Ki67 (proliferation marker).

The proliferative capacity of hair follicle cells serves as the fundamental cytological basis for hair growth, development, and follicle cycle maintenance, directly determining hair shaft growth rate, follicle structural integrity, and cycle progression [28,29]. Previous studies have demonstrated that DHT significantly upregulates the expression of androgen receptor (AR) in hair follicles, thereby inducing a growth-inhibitory state through suppression of follicle cell proliferation, which manifests as the typical pathological features of androgenetic alopecia. Furthermore, TGF-β1, as a key downstream effector molecule of the AR signaling pathway, is also upregulated upon DHT stimulation, synergistically exacerbating the proliferative inhibition of follicle cells (Figure 7A). To elucidate the impact of amCOF on follicular signaling pathways, the expression levels of AR, TGF-β1, and the proliferation marker Ki67 in mouse dorsal skin tissues were examined by immunofluorescence staining. The results revealed that, compared with the NC group, the AR fluorescence intensity in hair follicles of the model group was significantly elevated, accompanied by a consistent upregulation of TGF-β1 expression, whereas the proportion of Ki67-positive cells was markedly reduced (Figure 7B), indicating that DHT synergistically suppressed follicle cell proliferation through upregulation of AR and TGF-β1, thereby inducing a growth-inhibitory state. Following amCOF intervention, the expression levels of both AR and TGF-β1 were significantly downregulated, and the number of Ki67-positive cells substantially recovered (Figure 7C), suggesting that amCOF effectively reversed the DHT-induced pathological damage to hair follicles. The core mechanism of androgenetic alopecia involves the activation of downstream signaling cascades upon DHT binding to AR, among which TGF-β1, as a critical downstream effector, mediates cell cycle arrest and accelerates follicle miniaturization via the Smad signaling pathway, thereby amplifying growth-inhibitory signals. The synchronous upregulation of TGF-β1 and AR observed in the model group [30,31], along with their significant suppression after amCOF treatment, suggested that the therapeutic mechanism of amCOF might not be limited to physical adsorption of DHT but also involve the blockade of the AR/TGF-β1 pathogenic axis. Collectively, these results indicated that amCOF likely acted by adsorbing local DHT, reducing the formation of DHT–AR complexes, thereby blocking AR-mediated TGF-β1 upregulation and proliferative inhibition, restoring hair follicle cell proliferative activity, and improving follicular growth status, thus demonstrating promising potential in the prevention and treatment of AGA.

**Figure 8:**
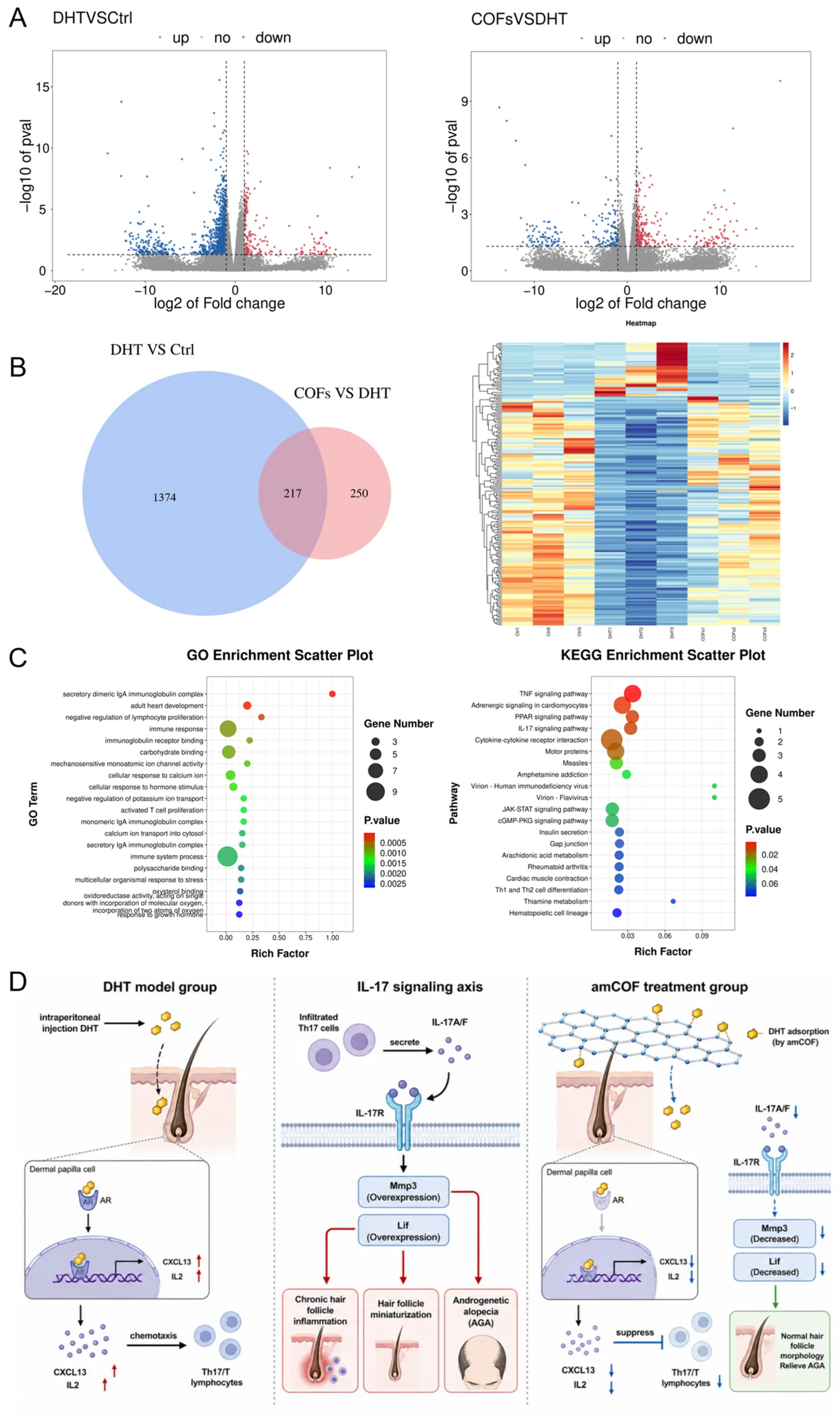
Transcriptome sequencing of different treatment groups: (**A**) Volcano plot of differentially expressed genes between the DHT-induced model group and the control group. Red and blue dots indicate significantly upregulated (*n* = 205) and downregulated (*n* = 1386) genes, respectively, based on a threshold of |log_2_ FC| ≥ 1 and *p* < 0.05. Gray dots represent genes with no significant change. Volcano plot of differentially expressed genes between the amCOF-treated group and the DHT-induced model group. Red and blue dots indicate significantly upregulated (*n* = 284) and downregulated (*n* = 183) genes, respectively, based on a threshold of |log_2_ FC| ≥ 1 and *p* < 0.05. Gray dots represent genes with no significant change. (**B**) Heatmap of differentially expressed genes across different treatment groups. Each column represents an individual sample, and each row represents a gene. Red and blue indicate high and low expression levels, respectively. The bar chart on the left shows the number of upregulated and downregulated genes in each comparison group. (**C**) Bubble plot of Gene Ontology (GO) and Kyoto Encyclopedia of Genes and Genomes (KEGG) pathway enrichment analysis of the differentially expressed genes. The size of each bubble represents the number of genes enriched in each term or pathway, and the color gradient indicates the statistical significance (*p*-value). The *x*-axis represents the enrichment factor, and the *y*-axis lists the enriched GO terms and KEGG pathways. (**D**) Signaling pathway network illustrating the potential mechanisms underlying the therapeutic effects of amCOF against AGA. The diagram summarizes the key pathways modulated by amCOF treatment, including the IL-17, which is involved in inflammatory responses and immune regulation during AGA progression.

Dfferentially expressed genes were identified in the DHT group, among which 205 genes were upregulated, and 1386 genes were downregulated. Following amCOF intervention, 467 differentially expressed genes were detected relative to the DHT group, with 284 genes upregulated and 183 genes downregulated, and 217 genes were commonly altered across all three groups (Figure 8A,B). GO functional enrichment analysis revealed that these differentially expressed genes were significantly enriched in immune-related biological processes, including immune response, immune system processes, and regulation of lymphocyte proliferation (Figure 8C). KEGG pathway analysis further demonstrated that the differentially expressed genes were predominantly enriched in inflammatory and immune regulatory pathways, such as the TNF signaling pathway, IL-17 signaling pathway, cytokine–cytokine receptor interaction, Th1 and Th2 cell differentiation, and JAK-STAT signaling pathway. Among these, the IL-17 signaling pathway exhibited the strongest association with DHT adsorption by amCOF and the reversal of AGA phenotypes. DHT accumulation significantly activated this pathway, with upregulation of pro-inflammatory genes, including Cxcl13 and Il2, which in turn promoted the infiltration of Th17 cells and activated T lymphocytes into the perifollicular region, leading to chronic micro-inflammation of hair follicles. Notably, amCOF treatment substantially reversed the aberrant activation of the IL-17 pathway and restored local cutaneous immune homeostasis (Figure 8D). Collectively, these transcriptomic findings provided molecular-level evidence that amCOF scavenges local DHT, thereby blocking androgen-triggered IL-17-mediated inflammatory cascades, alleviating perifollicular inflammatory damage, and ultimately suppressing AGA-associated hair follicle degeneration.

To evaluate the in vivo biosafety of amCOF after 14 days of topical application, which corresponds to the full duration of the therapeutic observation period, major organs, including the heart, liver, spleen, lung, and kidney, were collected from each group for histomorphological examination. H&E staining revealed that the organs in the NC group exhibited intact structures and normal cellular morphology, with no obvious pathological changes observed. Compared with the NC group, no apparent abnormalities such as edema, necrosis, or inflammatory cell infiltration were detected in any of the major organs in either the model group or the PC group. The amCOF-treated group also displayed clear tissue architecture and well-arranged cells, with no significant differences from the NC group (Figures S10 and S11), suggesting that amCOF exerted no obvious toxic effects on the major organs at the administered dosage and possessed favorable biocompatibility.

## 3. Methods

### 3.1. Synthesis of amCOF

2-[4-(dodecyloxy)phenyl]-1H-phenanthro[9,10-d]imidazole-5,10-diamine (PI-NH_2_, 0.075 mmol, 33.99 mg), 2,5-diaminobenzene-1,4-disulfonic acid (DABD, 0.045 mmol, 12.11 mg), and 4,4′,4″-(1,3,5-triazine-2,4,6-triyl)tribenzaldehyde (TFPT, 0.050 mmol, 19.52 mg) were sequentially added into a Pyrex pressure tube. Subsequently, 2.0 mL of a mixed solvent (*o*-dichlorobenzene/*n*-butanol, *v*/*v* = 1:1) was introduced into the tube, and the mixture was thoroughly stirred until homogeneous. Then, acetic acid (0.2 mL) was added as a catalyst. The resulting mixture was degassed by three freeze–pump–thaw cycles using liquid nitrogen to remove oxygen from the reaction system. The tube was then sealed and heated at 120 °C for 3 days to ensure complete reaction. After the reaction was completed, the product was washed repeatedly with organic solvents. Upon drying, the final product was obtained as a yellow powder of amCOF.

### 3.2. Contact Angle Measurement

Briefly, 150 mg of amCOF was dried under vacuum at 100 °C for 24 h to thoroughly remove residual solvents. Subsequently, 100 mg of the dried amCOF material was taken and pressed into a thin tablet with a smooth and flat surface using a tableting machine. The water contact angle and the oil contact angle on the surface of the tablet were then measured, respectively.

### 3.3. Effect of Contact Time on DHT Adsorption

A 20 mg·L^–1^ DHT standard solution was used as the adsorbate, and amCOF was employed as the adsorbent to investigate the effect of adsorption time on its adsorption performance. Accurately weighed 5.0 mg of amCOF was added to 10 mL of DHT standard solution with an initial concentration of 20 mg·L^–1^ at pH 7.0. The mixture was placed on a thermostatic magnetic stirrer and subjected to adsorption experiments at 25 °C with a stirring speed of 200 r·min^–1^. The adsorption times were set as 15, 30, 45, 60, 120, 180, and 240 min, respectively. Upon reaching the preset adsorption time, the adsorption mixture was immediately transferred to a centrifuge tube and centrifuged at 8000 r·min^–1^ for 2 min to thoroughly separate the amCOF material from the solution. The supernatant was collected and filtered through an organic nylon membrane filter (13 mm × 0.22 μm), and 5 mL of the filtrate was collected. The absorbance of the filtrate was measured using a UV–vis spectrophotometer, and the residual concentration of DHT in the solution was calculated according to the standard curve. The equilibrium adsorption capacity of amCOF toward DHT at time t was calculated by Equation (1).

(1) $$q_{t}=\frac{C_{0}-C_{t}}{m}\times V$$

Here, $q_{t}$ (mg·g^–1^) is the adsorption capacity of amCOF toward DHT at time *t*; $C_{0}$ (mg·L^–1^) is the initial concentration of DHT; $C_{t}$ (mg·L^–1^) is the residual concentration of DHT in the solution at time *t*; V (L) is the volume of the solution; and m (g) is the mass of amCOF used.

### 3.4. Effect of Adsorbent Dosage on DHT Adsorption

To evaluate the effect of adsorbent dosage, varying amounts of amCOF (2, 4, 5, 6, 8, 10, 12, and 14 mg) were added to separate conical flasks containing 100 mL of DHT solution (20 mg·L^–1^). The mixtures were stirred at 200 r·min^–1^ at 25 °C for 5 h to ensure equilibrium. After centrifugation (8000 r·min^–1^, 2 min), the supernatant was filtered through a 0.22 μm nylon membrane, and the absorbance was measured by UV–Vis spectrophotometry. A blank control without amCOF was processed in parallel under identical conditions to correct for non-adsorptive DHT loss.

(2) $$q_{e}=\frac{C_{0}-C_{e}}{m}\times V$$

Here, $q_{e}$ (mg·g^–1^) is the equilibrium adsorption capacity; $C_{0}$ (mg·L^–1^) is the initial concentration of DHT; $C_{e}$ (mg·L^–1^)is the residual concentration of DHT at adsorption equilibrium; *V* (L) is the volume of the solution; and *m* (g) is the mass of the amCOF adsorbent.

(3) $$R\%=\left[\frac{C_{0}-C_{t}}{C_{0}}\right]\times 100\%$$

Here, R is the DHT removal efficiency (%); $C_{0}$ (mg·L^–1^) is the initial concentration of DHT solution; $C_{t}$ (mg·L^–1^) is the residual concentration of DHT in solution at time t.

### 3.5. Adsorption Isotherms

The Langmuir model equation is expressed as follows:

(4) $$q_{e}=\frac{C_{e}q_{0}b}{C_{e}b+1}$$

where $C_{e}$ (mg·L^–1^) is the equilibrium concentration of DHT, b is the Langmuir constant, and $q_{0\;}$ (mg·g^–1^) is the maximum adsorption capacity.

The Freundlich model equation is expressed as follows:

(5) $$\ln q_{e}=\left(\frac{1}{n}\right)\ln C_{e}+\ln K_{f}$$

where $C_{e}$ (mg·L^–1^) is the equilibrium concentration of DHT, $K_{f}$ is the Freundlich adsorption equilibrium constant, $q_{e}$ (mg·g^–1^) is the equilibrium adsorption capacity, and *n* is an empirical constant reflecting the degree of influence of DHT concentration on adsorption capacity. The isothermal adsorption curve was fitted with $C_{e}$ and $q_{e}$ as the horizontal and vertical coordinates, respectively. The optimal adsorption model was determined according to the fitting coefficients of the Langmuir and Freundlich models.

### 3.6. Adsorption Kinetics

Based on the adsorption capacities calculated by Equation (1), the adsorption kinetics of amCOF toward DHT at different time intervals were obtained. To investigate the adsorption kinetic behavior of amCOF toward DHT, the pseudo-first-order kinetic model and the pseudo-second-order kinetic model were employed to fit the experimental data. The pseudo-first-order kinetic model was expressed as follows:

(6) $$\log\left(q_{\mathrm{eq}}-q_{t}\right)=\log q_{e}-\frac{k_{1}t}{2.303}$$

where $q_{t}$ (mg·g^−1^) and $q_{\mathrm{eq}}$ (mg·g^−1^) are the adsorption capacities of amCOF toward DHT at time *t* (min) and at equilibrium, respectively, and $K_{1}$ (min^−1^) is the pseudo-first-order rate constant.

The adsorption process of DHT onto the amphiphilic COF material was further analyzed using the pseudo-second-order kinetic model. Conformance to the pseudo-second-order model implies that the adsorption process involves electron transfer between the adsorbate and the adsorbent, which is characteristic of chemisorption. The pseudo-second-order kinetic model was expressed as follows:

(7) $$\frac{t}{q_{t}}=\frac{1}{k_{2}q_{\mathrm{eq}}^{2}}+\frac{t}{q_{\mathrm{eq}}}$$

where $q_{t}$ (mg·g^−1^) is the equilibrium adsorption capacity of amCOF toward DHT, and $K_{2}$ (g·mg^−1^·min^−1^) is the pseudo-second-order rate constant.

### 3.7. Cell Culture

Human dermal papilla cells (hDPCs) are from Zhejiang Meisen Cell Technology Co., Ltd. (Meisen CTCC, Jinhua, China). Dulbecco’s modified Eagle’s medium (DMEM) was prepared with 1% antibiotics and 10% fetal bovine serum. The cells were grown at 37 °C in a humidified 5% CO_2_ atmosphere [32].

### 3.8. CCK-8 Assay and Cell Migration Assay

The effect of amCOF on cell proliferation inhibited by DHT was evaluated using the CCK-8 assay (Solarbio, Beijing, China) [33]. hDPCs were seeded into a 96-well plate at 5 × 10^3^ cells/well and cultured for 24 h in DMEM. The concentrations of the treatment group (amCOF) and the DHT treatment group (Mod) used were 10 μg/mL and 1 μM, respectively. DMEM medium and Triton X-100 (Solarbio, Beijing, China) were used as negative control (NC) and blank, respectively. 10 μM minoxidil was used as positive control (PC). After treatment for 24 h, 10 μL of CCK-8 solution was added to each well, and the cells were then incubated at 37 °C for 1 h. The absorbance was measured at 450 nm by a multifunctional full-wavelength enzyme-labeled instrument (Varioskan LUX, Thermo Fisher Scientific, Waltham, MA, USA). Three technical replicate wells were used for each treatment condition within each independent experiment, and the entire experiment was repeated three times independently (biological replicates, *n* = 3).

In a 6-well plate, hDPCs were cultured at 1 × 10^5^ cells/well for 48 h until reaching confluent monolayers. Using a sterile 10 μL pipette tip, a straight scratch was made in the monolayer cells. Following gentle washing with sterile PBS, the cells were exposed to a serum-free medium. The concentrations of the treatment group (amCOF) and the DHT treatment group (Mod) were 1 μg/mL and 100 nM, respectively. Serum-free medium was used as a negative control (NC), and 10 μM minoxidil was used as a positive control (PC). Under an inverted microscope (Leica, Wetzlar, Germany), cell migration was monitored and imaged at 0–48 h. The migration distance was quantified using ImageJ software (1.8.0.345). Three independent experiments were performed, each with two technical replicate wells per condition.

### 3.9. C57BL/6 Mice Treatments

Six-week-old male *C57BL/6* mice were procured from Sipeifu Biotechnology Co., Ltd. (Beijing, China) and acclimatized for one week under specific-pathogen-free conditions (22 ± 2 °C, humidity 50 ± 5%, and light/dark cycle of 12 h). All animal operations were carried out in accordance with the ‘Guidelines for the Care and Use of Laboratory Animals of Changzhi Medical College’ and were approved by the Changzhi Medical College Experimental Animal Ethics Committee (DW2026039). After one week of acclimation, following random allocation, the mice were assigned to 4 groups, each consisting of 8 mice (*n* = 8): negative control (NC, topical application of physiological saline), model (Mod, intraperitoneal injection of DHT at 50 mg/kg), amCOF (topical application of 1 mg/mL and intraperitoneal injection of DHT at 50 mg/kg), and positive control (PC, topical application of 2% minoxidil and intraperitoneal injection of DHT at 50 mg/kg). Dorsal hair was shaved using scissors, and residual hair was removed using a depilatory cream (Veet, Chartres, France) one day before treatment. DHT was injected intraperitoneally. Physiological saline, amCOF, and 2% minoxidil were evenly applied to the back. No significant difference in food intake or body weight between groups was observed, and no adverse clinical signs occurred. After 15 days, the back skin was taken for HE staining and immunofluorescence staining. Animal Experiment Ethics Certification Approval Number: DW2026039.

### 3.10. Hair Regrowth Assessment

As the depilation on the back skin of *C57BL/6* mice induces hair follicles to enter the anagen [34,35], variations in depilation area on the dorsal skin were compared, and photographs were captured at regular intervals using a standardized camera setup. ImageJ software was utilized to quantify the hair regrowth using a four-point grading system reflecting progressive hair follicle activity based on dorsal skin color: 0 (pink skin, no hair regrowth), 1 (grey skin, initial hair growth), 2 (dark grey skin, advanced hair growth), and 3 (black skin, fully grown hair). The calculation for hair regrowth score was determined as follows:

Hair regrowth score = ((area of pink) × 0 + (area of grey) × 1 + (area of dark grey) × 2 + (area of black) × 3)/total area.

### 3.11. HE Staining

Mouse dorsal skin samples were fixed in 4% paraformaldehyde, paraffin-embedded, and cut into 6 μm sections. After dewaxing and rehydration, sections were stained with hematoxylin and eosin (Solarbio, Beijing, China), then dehydrated, cleared, and mounted to evaluate basic morphological characteristics among treatments under an optical microscope. The hair follicle size, number, and dermal thickness in the whole visual field were quantitatively analyzed by ImageJ software.

### 3.12. Immunofluorescence Staining

Paraffin sections with a thickness of 6 μm were dewaxed, washed, antigen-repaired, and serum-sealed for 30 min. The primary antibody (AR, 1:100; TGF-β1, 1:100; Ki67, 1:100) (Proteintech, Wuhan, China) was incubated at 4 °C overnight. After PBS washing, the fluorescent secondary antibody was incubated for 1 h at room temperature in the dark. After DAPI counterstaining of the nucleus, it was sealed using anti-fluorescence quenching, and the images were collected with a fluorescence microscope.

### 3.13. Statistical Analysis

All data are presented as mean ± standard deviation (SD) or standard error of the mean (SEM), as indicated in the figure legends. Statistical analyses were performed using GraphPad Prism 9.0 software (San Diego, CA, USA). Comparisons between two groups were analyzed using an unpaired two-tailed Student’s *t*-test. Comparisons among multiple groups were performed using one-way analysis of variance (ANOVA), followed by Tukey’s post hoc test. A value of *p* < 0.05 was considered statistically significant, with significance levels defined as * *p* < 0.05, ** *p* < 0.01. All experiments were performed with a minimum of three independent biological replicates, and each biological replicate contained at least three technical replicates.

## 4. Conclusions

In this study, a novel amphiphilic covalent organic framework (amCOF) was designed and synthesized to address the drug delivery bottleneck imposed by the sebum barrier in the treatment of androgenetic alopecia. The material possessed well-ordered mesoporous structures, favorable thermal stability, and a highly crystalline architecture maintained by an AA-stacking mode. Benefiting from the simultaneous incorporation of hydrophilic functional groups and lipophilic alkyl chains into the framework, amCOF exhibited excellent amphiphilic properties and achieved highly efficient and selective adsorption of DHT in a simulated sebum–water interface environment, with an equilibrium adsorption capacity of 118.45 mg·g^−1^. In vitro cellular assays confirmed that amCOF effectively eliminated DHT from the follicular microenvironment and reversed its inhibitory effects on the proliferation and migration of dermal papilla cells. In vivo animal experiments further demonstrated that topical application of amCOF significantly reduced local DHT concentrations and decreased the formation of DHT–AR complexes, thereby blocking AR-mediated TGF-β1 upregulation and proliferative inhibition, restoring the proliferative activity of hair follicle cells, and effectively improving follicular growth status. Notably, no obvious adverse effects were observed, indicating favorable biocompatibility. This strategy not only provides a novel material design concept for overcoming the sebum barrier but also establishes a solid foundation for expanding the biomedical applications of covalent organic frameworks in the treatment of skin-related disorders.

## Data Availability

The original contributions presented in this study are included in the article/Supplementary Materials. Further inquiries can be directed to the corresponding authors.

## Supplementary Materials

The following supporting information can be downloaded at https://www.mdpi.com/article/10.3390/molecules31162936/s1, Figure S1: ^1^H NMR spectra of TFPT (600 MHz, DMSO); Figure S2: ^13^C NMR spectra of TFPT (600 MHz, DMSO); Figure S3: ^1^H NMR spectra of PI-NH_2_ (600 MHz, DMSO); Figure S4: ^13^C NMR spectra of PI-NH_2_ (150 MHz, DMSO); Figure S5: Ultraviolet absorbance and standard curve of dihydrotestosterone (DHT); Figure S6: Langmuir isotherm fitting for DHT adsorption onto amCOF; Figure S7: Freundlich isotherm fitting for DHT adsorption onto amCOF; Figure S8: Hair follicle size statistics in different treatment groups; Figure S9: Hair follicle number in different treatment groups; Figure S10: Cytocompatibility of amCOF compared with PBS; Figure S11: H&E staining of major organs from different treatment groups.
