# Supplementary material for: Amphiphilic Covalent Organic Framework for Efficient DHT Adsorption and Androgenetic Alopecia Treatment via Spectral Technology
Source: Molecules. 2026 Aug 21;31(16):2936. doi: 10.3390/molecules31162936 (PMC13517036; doi:10.3390/molecules31162936)
Supplement: Supplementary file 1 [file molecules-31-02936-s001.zip › molecules-4473554-supplementary.pdf]

Supporting Information

Amphiphilic Covalent Organic Framework for Efficient DHT Adsorption and Androgenetic Alopecia Treatment via Spectral Technology

Jiahui Wei [1], Fanqiang Bu [1,*], Bing Zhao [1, *], Qi Liu [a], and Jinqiang Wu [2, *]

[1] *Heilongjiang Provincial Key Laboratory of Surface Active Agent and Auxiliary, Chemistry and Chemical Engineering Institute, Qiqihar University, Qiqihar 161006, China*

[2] *School of Basic Medical Sciences, Changzhi Medical College, Changzhi, Shanxi, 046000, China*

* Corresponding authors. *E-mail address*: bufq@qqhru.edu.cn (Fanqiang Bu), qqhrzhao@qqhru.edu.cn (Bing Zhao),and wujinqiang@czmc.edu.cn (Jinqiang Wu).

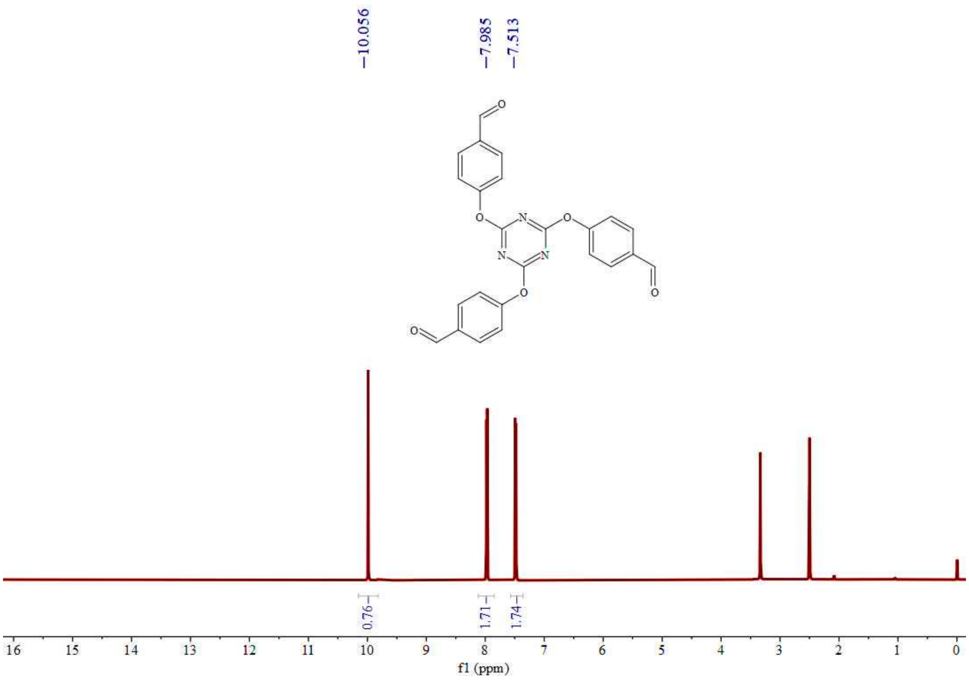

Figure S1 ^{1}H NMR spectra of TFPT (600 MHz, DMSO).

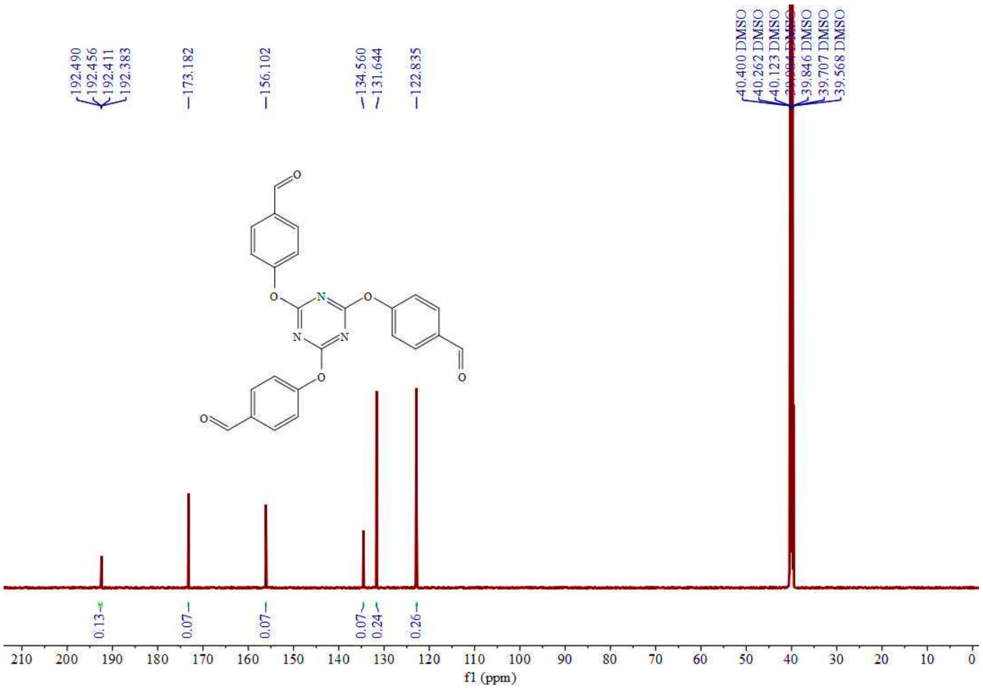

Figure S2 ^{13}C NMR spectra of TFPT (600 MHz, DMSO).

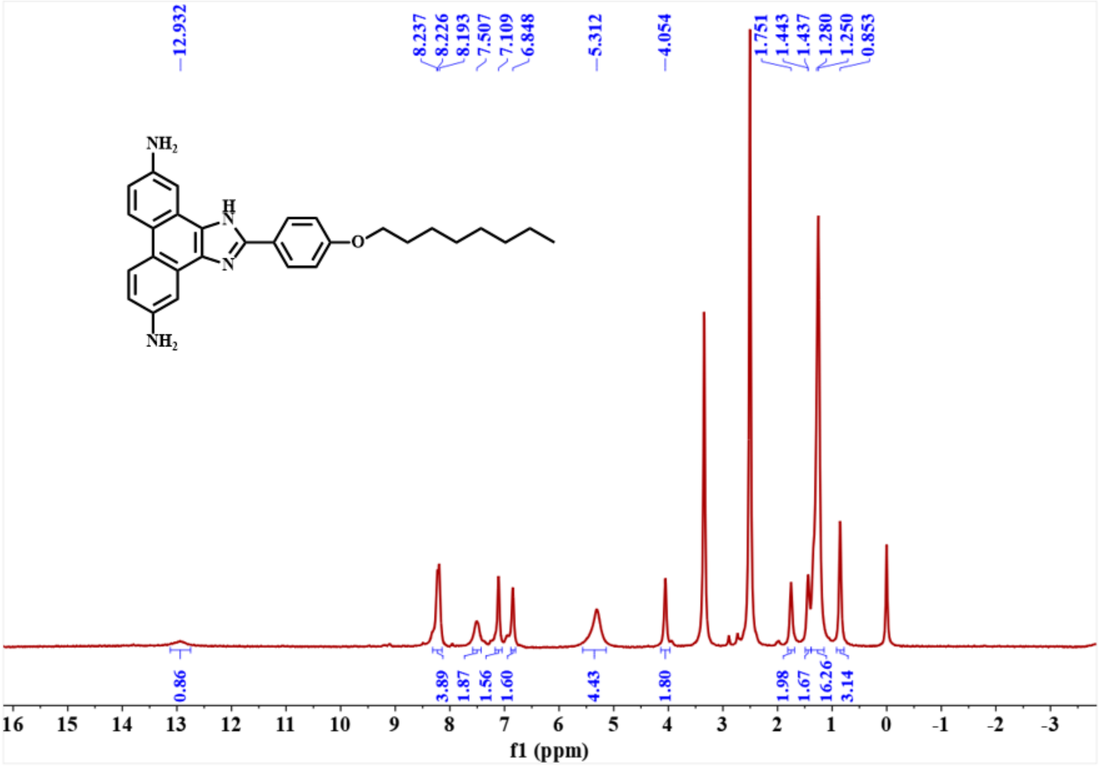

Figure S3 ^{1}H NMR spectra of PI-NH_2 (600 MHz, DMSO).

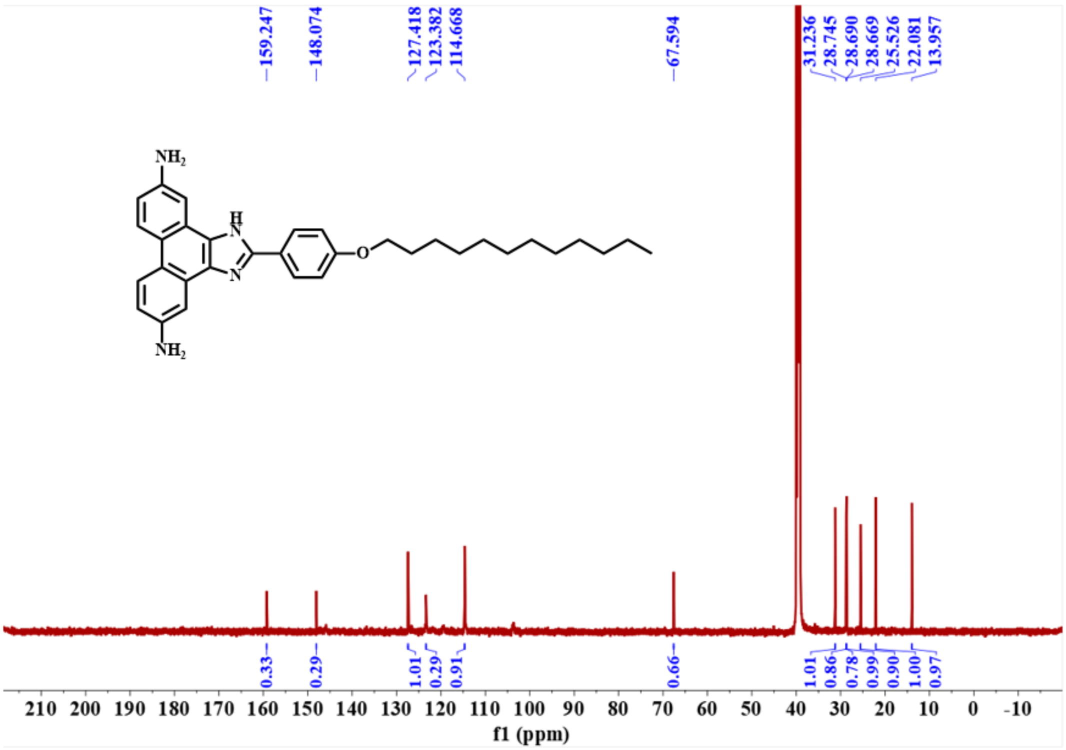

Figure S4 ^{13}C NMR spectra of PI-NH_2 (150 MHz, DMSO).

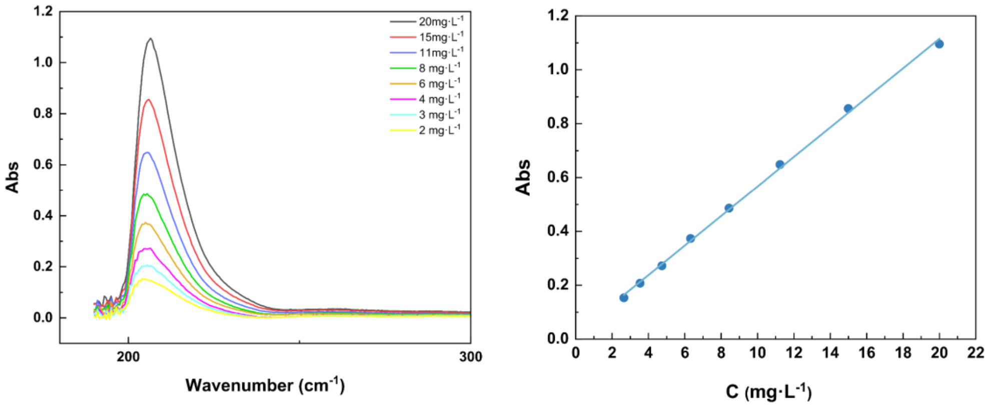

Figure S5 Ultraviolet absorbance and standard curve of dihydrotestosterone (DHT)

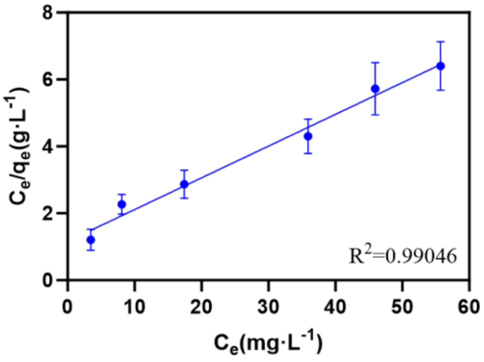

Figure S6 Langmuir isotherm fitting for DHT adsorption onto amCOF.

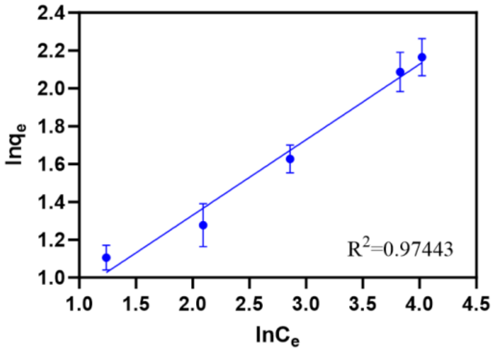

Figure S7 Freundlich isotherm fitting for DHT adsorption onto amCOF.

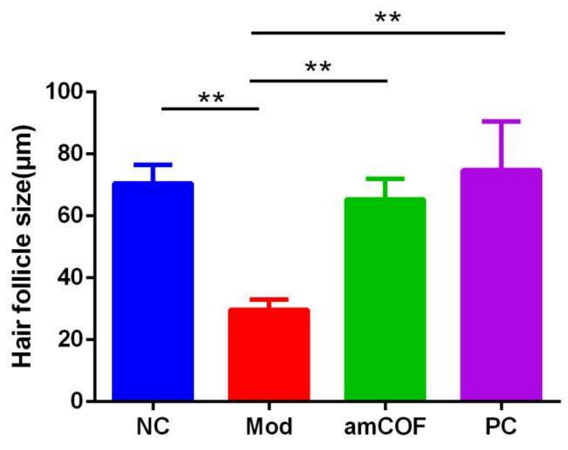

Figure S8 Hair follicle size statistics in different treatment groups

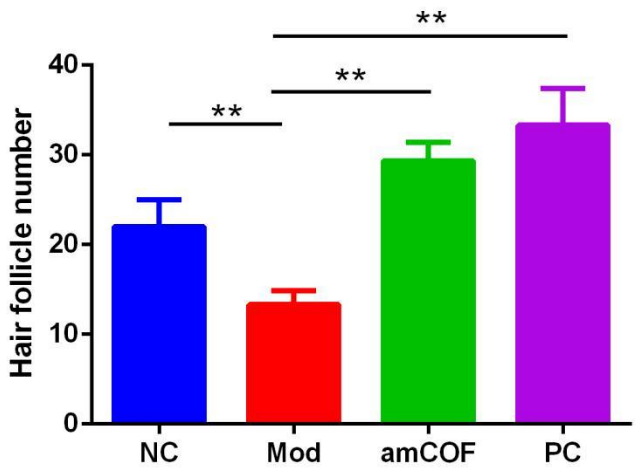

Figure S9 Hair follicle number in different treatment groups.

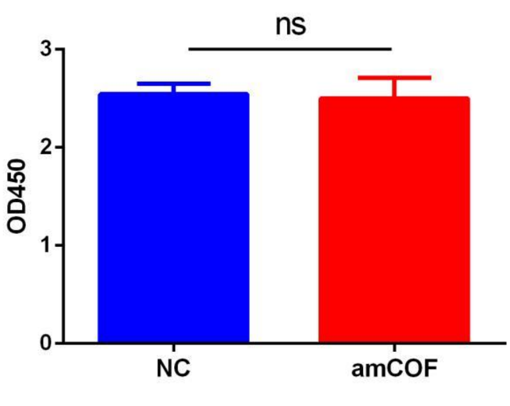

Figure S10 Cytocompatibility of amCOF compared with PBS

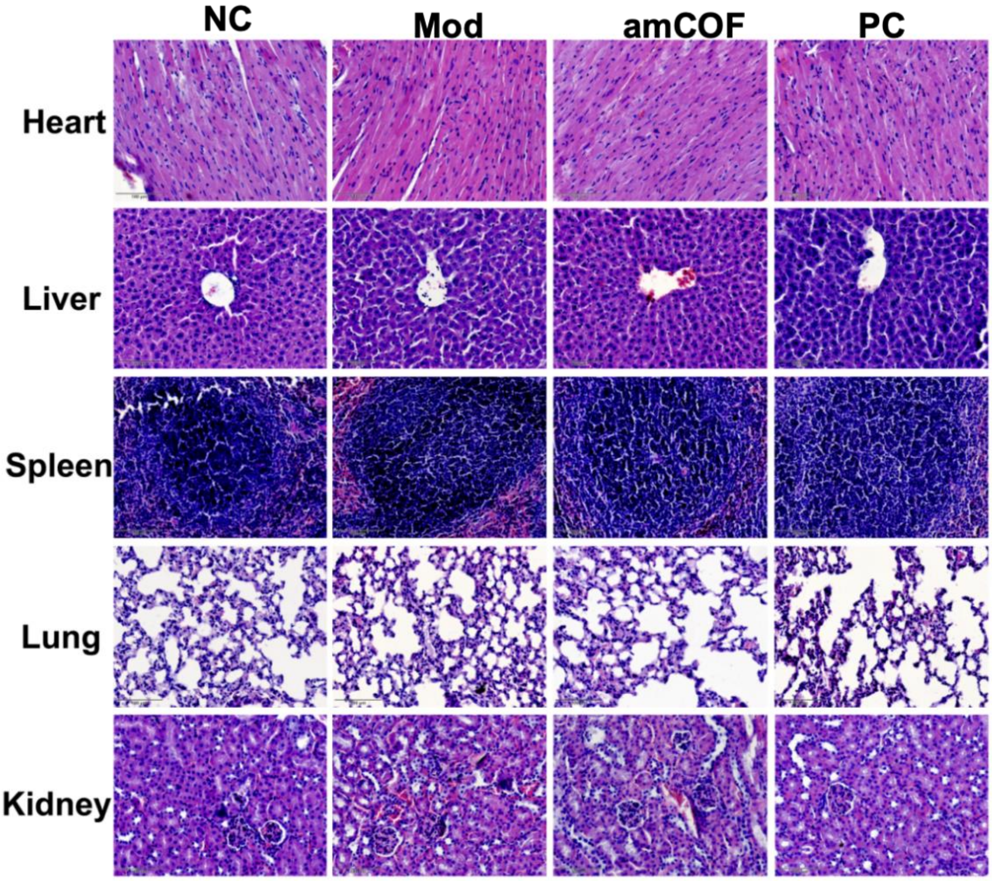

Figure S11 H&E staining of major organs from different treatment groups.
